# Supplementary material for: Effects of Renal Denervation on the Enhanced Renal Vascular Responsiveness to Angiotensin II in High-Output Heart Failure: Angiotensin II Receptor Binding Assessment and Functional Studies in Ren-2 Transgenic Hypertensive Rats
Source: Biomedicines. 2021 Nov 30;9(12):1803. doi: 10.3390/biomedicines9121803 (PMC8698780; doi:10.3390/biomedicines9121803)
Supplement: Supplementary file 1 [file biomedicines-09-01803-s001.zip › biomedicines-1411280-supplementary.pdf]

Supporting document to:

Effects of renal denervation on the enhanced renal vascular responsiveness to angiotensin II in high-output heart failure: angiotensin II receptor binding assessment and functional studies in Ren-2 transgenic hypertensive rats

Preparation of [^{125}I]-[Sar1, Ile8]-Angiotensin II

Radiolabeled [^{125}I]-[Sar1, Ile8]-Angiotensin II was prepared via radioiodination of Tyr of [Sar1, Ile8]-Angiotensin II with ^{125}I (Na[^{125}I], product code: I-RB-41, 2 mCi, IZOTOP, Hungary), using the IODO-GEN™ system (Pierce) [1,2]. An Eppendorf PP tube (1.5 mL), pre-coated in house with 10 nmoles of IODO-GEN™ (Pierce), was rinsed with 200 μL of PBS (0.1 M phosphate buffer pH 7.6, 0.15 M NaCl). Fresh PBS (200 μL), sodium iodide (20 μL Na[^{125}I], 2 mCi) and aqueous solution 80 μL of [Sar1, Ile8]-angiotensin II (1 μg/μL, 80 nmol) were mixed in the reaction tube. The tube was vigorously shaken for 15 min at room temperature and then 200 μL of 20 mM HEPES buffer (pH 7.3) containing BSA (1 mg/mL) was added. BSA was used to suppress the stickiness of the peptide to the reaction tube walls. The mixture was directly injected into the semipreparative radio-HPLC system (Waters Alliance e2695, 2995 PDA detector and Empower 3 software for processing of data) combined with radioactivity-HPLC flow detector Ramona Star (Elysia-Raytest, Germany). The desired [^{125}I]-[Sar1, Ile8]-Angiotensin II was separated from the unmodified and over-iodinated analog on Synergi POLAR column (5μ, 250 × 4.6 mm, Phenomenex) at 25 °C. The mobile phases were: A, 0.1 % (v/v) TFA in 100 % water and B, 0.1 % (v/v) TFA in 100 % acetonitrile. The flow rate was 1 mL/min. The gradient started at 10 % mobile phase B, increased linearly to 40 % mobile phase B over 50 min. Fractions were collected to tubes with 200 μL of BSA cocktail (20 mM HEPES; pH 7.3; BSA 1 mg/mL). The isolated fractions containing [^{125}I]-monoiodotyrosyl-[Sar1,Ile8]-Angiotensin II were transferred to LoBind® Eppendorf tubes and evaporated to dryness on CentriVap at room temperature for 4 hours. Radioactivity was measured using the Gamma Counter Wizard^{2} 2470 (Perkin Elmer). The retention time of the unmodified analog was 15 min. The desired mono-iodinated product at Tyr had a retention time of 18 min. We finally obtained 1350 μCi (50 MBq) of mono-iodinated [Sar1,Ile8]-Angiotensin II (67 % RCY, >99 % RCP). Radio-chromatogram of purification of [^{125}I]-[Sar1,Ile8]-Angiotensin II is shown in Figure S1.

Equipment: Analytical-semipreparative radio-HPLC (Waters Alliance e2695, 2995 PDA detector and Empower 3 software for processing of data) combined with radioactivity-HPLC flow detector Ramona Star (Elysia-Raytest). The Gamma Counter Wizard² 2470 (Perkin Elmer) for quantification of γ-ionization.

Supplemental References:

1. Elbert T., Vesela I., Conditions for successful labeling of oxidation sensitive peptides by Na[I-125]-IODO-GEN™ system, *J Label Compd Radiopharm*. **2010**, 53, 288–299. https://doi.org/10.1002/jlcr.1765
2. Jiracek J., Zakova L., Marek A., Radiolabeled hormones in insulin research, a minireview., *J Label Compd Radiopharm*. **2020**, 63, 576–581. https://doi.org/10.1002/jlcr.3881

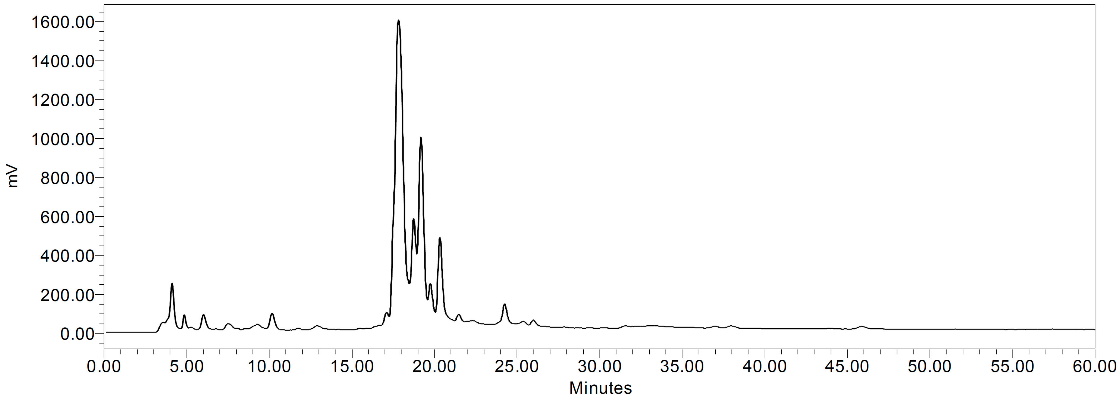

Figure S1: Radio-chromatogram of purification of [^{125}I]-[Sar1,Ile8]-Angiotensin II
